# *Fasciolopsis buski* Detected in Humans in Bihar and Pigs in Assam, India 

**DOI:** 10.3201/eid2806.220171

**Published:** 2022-06

**Authors:** Dipshikha Saikia, Yugal K. Prasad, Suman Dahal, Sudeep Ghatani

**Affiliations:** Sikkim University, Gangtok, India (D. Saikia, S. Dahal, S. Ghatani);; Shri Shubh Lal Hospital and Research Centre, Sitamarhi, India (Y.K. Prasad)

**Keywords:** food safety, fasciolopsiasis, Fasciolopsis buski, foodborne illnesses, foodborne trematode, parasites, enteric infections, zoonoses, India

## Abstract

The foodborne intestinal trematode *Fasciolopsis buski* causes the neglected zoonotic disease fasciolopsiasis. We detected *F. buski* infection in 14 pediatric patients in Sitamarhi, Bihar, and in pigs in Sivasagar, Assam, India. Proper diagnostic methods and surveillance are urgently needed to accurately estimate the true burden of this disease in India.

*Fasciolopsis buski* is a foodborne intestinal trematode that causes the neglected zoonotic disease fasciolopsiasis in humans and pigs. *F. buski* infection is transmitted through ingestion of raw aquatic plants or water carrying encysted metacercaria. Persons with substantial worm loads can have clinical indicators, such as malnutrition, edema, malabsorption, severe diarrhea, ascites, and anemia, and might experience acute intestinal obstruction and ileus (*1*–*3*). *F. buski* worms are found mostly in Asia and the Indian subcontinent; endemicity is highest in eastern India (*4*). We previously reported multiple cases of infection with *Artyfechinostomum sufrartyfex*, an echinostome trematode, which was diagnosed in children at Shri Shubh Lal (SSL) Hospital and Research Centre in Sitamarhi, Bihar state, India (*5*). We also documented several cases of fasciolopsiasis among SSL patients during 2012–2021 and infection in pigs detected in Sivasagar district, Assam state, India during a 2019–2020 survey. 

Infections with this parasite have been reported from diverse regions of India, as well as other parts of Asia (*6*–*10*). A genetic study suggested that the species found in India is different from species found in China and Vietnam (*11*). To corroborate the genetic distinctions between the strains found in India and those from China and Vietnam, we determined the complete nuclear ribosomal ITS2 and partial mitochondrial cytochrome c oxidase subunit 1 gene (cox1) sequences of *F. buski* from the samples recovered from Bihar and Assam and compared them to sequences from isolates from other regions of India, China, and Vietnam. The institutional ethics committee of Sikkim University in Gangtok, India, approved this work (SU/REG/F-1/03/2018/VOL-1/59). 

## The Study

During 2012–2021, a total of 14 children 3–12 years of age were brought for treatment to SSL Hospital for reported loose bowel movements, including watery feces and feces tinged with blood and mucus for >15 days, as well as vomiting, flatulence, abdominal discomfort, pain in the abdomen, fever, loss of appetite, weakness, and passage of flat reddish worms, called paterwa or lal keera in local languages. Eight patients were male and 6 female. Among the male patients, 3 were ≤5, 3 were 6–10, and 2 were >10 years of age; among the female patients, 3 were ≤5 and 3 were ≥10 years of age. All of the patients were of low socioeconomic status and resided near ponds or deep-water rice paddies contaminated with human and animal excreta and snail-infested areas. The patients were habituated to consume raw snails, contaminated water, chestnuts, and vegetables irrigated with contaminated water from nearby ditches.

On physical examination, all patients were pale and malnourished. General and systemic examination revealed persistent diarrhea, dehydration, and vomiting in most and anemia in all of the case-patients. Laboratory investigation revealed most of the patients had eosinophilia, and grade II malnutrition was associated with most patients (Table 1). All patients tested negative on tuberculin and HIV tests, and results from routine urine examination, complete blood counts, serum urea, serum creatinine, serum bilirubin, and alanine aminotransferase testing were within reference ranges (Appendix 1). 

**Table 1:** General and clinical symptoms for 14 patients with *Fasciolopsis buski* infections recorded in SSL Hospital, Bihar, India

Category	No. cases	Test value
Sign or symptom from general and systemic examination		
Persistent diarrhea	13	Y
Acute diarrhea	1	Y
Dehydration	12	Y
Abdomen pain	5	Y
Passage of live worms in feces or vomitus	2	Y
Passage of dead worms in feces or vomitus	12	Y
Fever	9	Y
Vomiting	12	Y
Anemia	14	10.9–14.1 g/dL*
Eosinophilia	10	50–500 eosinophils/mm^3^ (1%–4%)
Total leukocyte count	7	5,000–10,000 leukocytes/μL of blood
Potassium	1	3.4–4.7 mEq/L
Malnutrition grading		
Malnutrition grade I, mild malnutrition	1	71%–80%
Malnutrition grade II, moderate malnutrition	9	61%–70%
Malnutrition grade III, severe malnutrition	4	51%–60%
*Children 3–12 years of age.		

Naked eye examination of the feces revealed the presence of live parasites in 2 patients and dead parasites in 12. Two patients had mixed infection: 1 with both *F. buski* and *A. sufrartyfex* parasites and the other with *F. buski* worms and the roundworm *Ascaris* *lumbricoides*. On microscopic examination of feces samples, no eggs or ova of intestinal flukes were identified, except 1 sample showed fertilized roundworm ova. We isolated the recovered parasites and processed them for morphologic, anatomic, and genetic analysis. All of the patients were hospitalized and treated with praziquantel (75 mg/kg bodyweight; 3 divided doses for 2 d) and supportive measures administered for dehydration, electrolyte imbalance, and malnutrition. All patients were cured and discharged after being counseled for nutritional rehabilitation. 

**Figure 1:**
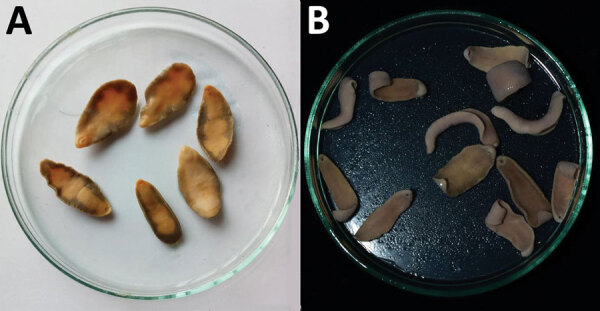
*Fasciolopsis buski* trematode samples preserved in absolute ethanol. A) Parasites recovered from child patients in Shri Shubh Lal Hospital and Research Centre Hospital, Sitamarhi, Bihar, India. B) Parasites isolated from the intestine of freshly slaughtered pigs in Sivasagar district of Assam.

In 2019–2020, a survey of pigs for *F. buski* infection was performed in the Charaideo, Sivasagar, Lakhimpur, Biswanath, and Tezpur districts of Assam. A total of 128 pigs were examined; 3 in the Sivasagar district displayed evidence of parasite infection. The flukes were collected in 0.9% phosphate-buffered saline (pH 7.2) from the intestines of freshly slaughtered pigs in Sivasagar district, as well as from the feces and vomitus of SSL Hospital patients, and brought to the Sikkim University Department of Zoology for further analysis (Figure 1). 

We extracted and purified genomic DNA from the flukes collected from both patients and pigs using QIAGEN DNeasy Blood and Tissue Kit (https://www.qiagen.com) according to manufacturer instructions. We performed amplification and sequencing of the complete ITS2 and partial cox1 genes using the primers 3S: 5′-GGTACCGGTGGATCACTCGGCTCGTG-3′ (forward), A28: 5′-GGGATCCTGGTTAGTTTCTTTTCCTCCGC-3′ (reverse) (*12*,*13*), and DICE 1F: 5′-TTWCNTTRGATCATAAG, Dice 14R: 5′-CCHACMRTAAACATATGATG-3′ (reverse) (*14*). The ITS2 amplicon was ≈292 bp and the cox1, ≈784 bp. We uploaded sequences to GenBank (ITS2 accession nos. MW771525 [Sitamarhi] and MW771526 [Sivasagar]; cox1 accession nos. MW767135 [Sitamarhi] and MW767136 [Sivasagar]. We identified parasites using a BLASTn search (https://blast.ncbi.nlm.nih.gov/Blast.cgi). 

**Figure 2:**
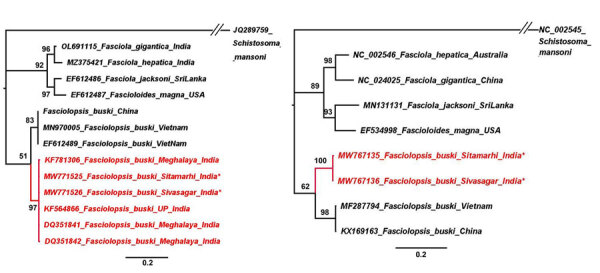
Phylogenetic trees for study strains of *Fasciolopsis buski* trematodes recovered from child patients in Sitamarhi, Bihar, and pigs in Sivasagar, Assam, India, and reference sequences. Red indicates isolates from India; asterisks indicate strains from this study. Tree was constructed using the maximum-likelihood method as implemented in MrBayes version 3.1.2 (https://bioweb.pasteur.fr/packages/pack@mrbayes@3.1.2). A) Internal transcribed spacer 2 gene tree using Hasegawa-Kishino-Yano plus invariate sites model. B) Cytochrome c oxidase subunit 1 gene tree using general time reversible plus gamma model. The analyses were run for 5,000,000 generations with sampling frequency of 100 and initial 25% of the trees discarded as burn-in. Node values represent Bayesian posterior probabilities. GenBank accession numbers are provided when available. Scale bars represent branch length.

The ITS2 sequences of the Sitamarhi and Sivasagar isolates were genetically similar and showed the greatest sequence similarity with previously identified *F. buski* isolates from Uttar Pradesh (GenBank accession no. KF564866) and Meghalaya, India (accession no. DQ351841), with little or no genetic variability. In contrast, *F. buski* sequences from China and Vietnam (GenBank accession nos. MN970005 and EF612489) had 7.7%–8.2% genetic difference from the isolates from India. However, the sequences from China and Vietnam were identical to each other. Similarly, the cox1 sequences from Sitamarhi and Sivasagar exhibited only 0.4% variation between each other but 12.1%–12.3% variation from sequences from Vietnam (accession no. MF287794) and China (accession no. KX169163). The sequences from China and Vietnam had only 0.5% variation from each other (Table 2; Appendix 2 Figures 1, 2). 

**Table 2:** Genetic variations in *Fasciolopsis buski* ITS2 and COI gene regions from India and other Asian countries*

Gene region	Genetic distance							
	Among Indian isolates			From other Asian isolates†			Among other Asian isolates†	
	% Isolates	Transitions/ transversions		%	Transitions/ transversions		%	Transitions/ transversions
Nuclear ribosomal ITS2	0–0.3	1/0		7.7–8.2	12/8		0	0/0
Mitochondrial COI	0.4	1/0		12.1–12.3	36/46		0.5	3/1
*COI, cytochrome oxidase subunit 1; ITS2, internal transcribed spacer 2 †Other Asian isolates from China and Vietnam								

We constructed phylogenetic trees based on the 2 gene regions using the maximum-likelihood method. Both trees clearly showed the Indian isolates forming a separate clade from the isolates from China and Vietnam (Figure 2). On the basis of our findings, we concluded that the samples collected in our study belonged to *F. buski* but that the isolates from China and Vietnam were separate taxa from those from India; however, *F. buski* samples from China and Vietnam were the same species. This discovery is consistent with the findings of a prior study in China (*11*).

## Conclusions

Our study confirmed that the parasites obtained from both human patients in Sitamarhi and pigs in Sivasagar were of the *F. buski* species. However, we also corroborated that the species found in India might differ from those in China and Vietnam, and species taxonomy might need to be revised in the future (*12*). 

In recent years, *F. buski* infection from humans and pigs has been documented in India in the states of Assam, Bihar, Delhi, Meghalaya, and Uttar Pradesh (*13*). According to our findings, this parasite is an increasing public health threat in India, especially in remote locations and among persons from low socioeconomic backgrounds, because of the substantial risk to human and animal health it poses. Surveys are urgently needed to determine the true burden of fasciolopsiasis in the country. A lack of effective diagnostic tools for detecting neglected foodborne trematode infections, including *F. buski*, means there are no prevalence data on these infections in the country. Therefore, there is a pressing need to design and develop a rapid and easy detection tool for *F. buski* and other neglected trematode infections (*14*). 

Appendix 1 Additional patient demographics and details from routine laboratory investigation reports in study of *Fasciolopsis buski* in humans and pigs in eastern India.

Appendix 2 Additional details of gene sequencing from study of *Fasciolopsis buski* in humans and pigs in eastern India.
