# Supplementary material for: Fasciolopsis buski Detected in Humans in Bihar and Pigs in Assam, India
Source: Emerg Infect Dis. 2022 Jun;28(6):1265–8. doi: 10.3201/eid2806.220171 (PMC9155878; doi:10.3201/eid2806.220171)
Supplement: Appendix 2 — Additional details of gene sequencing from study of Fasciolopsis buski in humans and pigs in eastern India. [file 22-0171-Techapp-s2.pdf]

Article DOI: https://doi.org/10.3201/eid2806.220171

Fasciolopsis buski Detected in Humans in Bihar and Pigs in Assam, India

Appendix 2

```
                          ....|....| ....|....| ....|....| ....|....| ....|....| ....|....|
                                  10         20         30         40         50         60
MF287794_Vietnam          DHKRIGFVYL VVGLWSGFLG LSLSMLIRLN YLDPYYNLIS PEVYNYLVTG HGIVMIFFFL
KX169163_China            .......... .......... .......... .......... .......... ..........
MW767135_Sitamarhi_India  .....C.I.. I......... .......... .......... .......... ..........
MW767136_Sivasagar_India  .......I.. .......... .......... .......... .......... ..........

                          ....|....| ....|....| ....|....| ....|....| ....|....| ....|....|
                                  70         80         90        100        110        120
MF287794_Vietnam          MPVLIGGFGN YLLPLLLGIG DLNLPRVNAL SVWLLLPSCV CLALSLIGGA GVGWTFYPPL
KX169163_China            .......... .......... .......... .......... .......... ..........
MW767135_Sitamarhi_India  .......... .......... ......I... ........S. ......L... ..........
MW767136_Sivasagar_India  .......... .......... ......I... ........S. ......L... ..........

                          ....|....| ....|....| ....|....| ....|....| ....|....| ....|....|
                                 130        140        150        160        170        180
MF287794_Vietnam          SASGYSGWGV DFMMFSLHLA GVSSIFSSIN FICTIVEVMF EEGTGRLSIL VWAYLFTSIL
KX169163_China            .......... .......... .......... .......... .......... ..........
MW767135_Sitamarhi_India  .SF....... ..L....... .......... .......... .......... ..........
MW767136_Sivasagar_India  .SF....... ..L....... .......... .......... .......... ..........

                          ....|....| ....|....| ....|....| ....|....| ....|....| ....|....|
                                 190        200        210        220        230        240
MF287794_Vietnam          LLLSLPVLAA AITMLLFDRS FGSAFFDPMG GGDPVLFQHL FWFFGHPEVY VLILPGFGVI
KX169163_China            .......... .......... .......... .......... .......... ..........
MW767135_Sitamarhi_India  .......... .......... .......... .......... .......... ..........
MW767136_Sivasagar_India  .......... .......... .......... .......... .......... ..........

                          ....|....| ....|....| ..
                                 250        260
MF287794_Vietnam          SHICVTLTNN DSLFGYYGLV LX
KX169163_China            .......... .......... ..
MW767135_Sitamarhi_India  .......... .......... ..
MW767136_Sivasagar_India  .......... .......... ..
```

Appendix 2 Figure 1. Alignment of the ITS2 gene region highlighting genetic variations (red) between *F. buski* isolates from India and from China and Vietnam.

```
                          ....|....| ....|....| ....|....| ....|....| ....|....| ....|....|
                                  10         20         30         40         50         60
MN970005_Vietnam          GCTTATAAAC TATCACGACG CCCAAAAAGT CGTGGCTTGG GTTTTGCCAG CTGGCGTGAT
EF612489_Vietnam          .......... .......... .......... .......... .......... ..........
China                     ---....... .......... .......... .......... .......... ..........
DQ351842_Meghalaya_India  .....C.... .......... .......... .......... ..C....... .........A
MW771525_Sitamarhi_India  .....C.... .......... .......... .......... ..C....... .........A
MW771526_Sivasagar_India  .....C.... .......... .......... .......... ..C....... .........A
KF564866_UP_India         .....C.... .......... .......... .......... ..C....... .........A
DQ351841_Meghalaya_India  .....C.... .......... .......... .......... ..C....... .........A
KF781306_Meghalaya_India  .....C.... .......... .......... .......... ..C....... ....-C...A

                          ....|....| ....|....| ....|....| ....|....| ....|....| ....|....|
                                  70         80         90        100        110        120
MN970005_Vietnam          TTCCTCTATG ATTATTCATG TGAGGTGCCA GAACTATGGC GTTTCCCTAA TGTATCCGGA
EF612489_Vietnam          .......... .......... .......... .......... .......... ..........
China                     .......... .......... .......... .......... .......... ..........
DQ351842_Meghalaya_India  C......... .......... .......... .......... .......... ..........
MW771525_Sitamarhi_India  C......... .......... .......... .......... .......... ..........
MW771526_Sivasagar_India  C......... .......... .......... .......... .......... ..........
KF564866_UP_India         C......... .......... .......... .......... .......... ..........
DQ351841_Meghalaya_India  C......... .......... .......... .......... .......... ..........
KF781306_Meghalaya_India  C......... .......... .......... .......... .......... ..........

                          ....|....| ....|....| ....|....| ....|....| ....|....| ....|....|
                                 130        140        150        160        170        180
MN970005_Vietnam          CGCGTCCTTG TCTCGGCAGA AGGCTGTGAT GAGGTGCGGT AGCGGAATCG TGGTCTAATG
EF612489_Vietnam          .......... .......... .......... .......... .......... ..........
China                     .......... .......... .......... .......... .......... ..........
DQ351842_Meghalaya_India  .......... ....A..... ........G. .......... .....G.... ....T.....
MW771525_Sitamarhi_India  .......... ....A..... ........G. .......... .....G.... ....T.....
MW771526_Sivasagar_India  .......... ....A..... ........G. .......... .....G.... ....T.....
KF564866_UP_India         .......... ....A..... ........G. .......... .....G.... ....T.....
DQ351841_Meghalaya_India  .......... ....A..... ........G. .......... .....G.... ....T.....
KF781306_Meghalaya_India  .......... ....A..... ........G. .......... .....G.... ....T.....

                          ....|....| ....|....| ....|....| ....|....| ....|....| ....|....|
                                 190        200        210        220        230        240
MN970005_Vietnam          ATTACTGTGC ACGTTCTGCT GCTGTGTCCT CATCGTCGGC TTGATGCGAG ATTAGGTATC
EF612489_Vietnam          .......... .......... .......... .......... .......... ..........
China                     .......... .......... .......... .......... .......... ..........
DQ351842_Meghalaya_India  .A........ ......C.T. ........T. .......... ........T. .C.T...C..
MW771525_Sitamarhi_India  .A........ ......C.T. ........T. .......... ........T. .C.T...C..
MW771526_Sivasagar_India  .A........ ......C.T. ........T. .......... ........T. .C.T...C..
KF564866_UP_India         .A........ ......C.T. ........T. .......... ........T. .C.T...C..
DQ351841_Meghalaya_India  .A........ ......C.T. ........T. .......... ........T. .C.T...C..
KF781306_Meghalaya_India  .A........ ....-.C.T. ........T. .......... ........T. .C.T...C..

                          ....|....| ....|....| ....|....| ....|....| ....|....| ..
                                 250        260        270        280        290
MN970005_Vietnam          GTGTCTGAGG CTTTTGCCAT ACACAGACTG CAAATTTTGT GGTCTAATTT CC
EF612489_Vietnam          .......... .......... .......... .......... .......... ..
China                     .......... .......... .......... .......... .......... ..
DQ351842_Meghalaya_India  .......... .......... ...T.A.... .C.T..G... .......... ..
MW771525_Sitamarhi_India  .......... .......... ...T...... .C.T..G... .......... ..
MW771526_Sivasagar_India  .......... .......... ...T...... .C.T..G... .......... ..
KF564866_UP_India         .......... .......... ...T...... .C.T..G... .......... ..
DQ351841_Meghalaya_India  .......... .......... ...T...... .C.T..G... .......... ..
KF781306_Meghalaya_India  .......... .......... ...T...... .C.T..G... .......... ..
```

Appendix 2 Figure 2. Alignment of the cox1 gene region highlighting genetic variations (red) between *F. buski* isolates from India and from China and Vietnam.

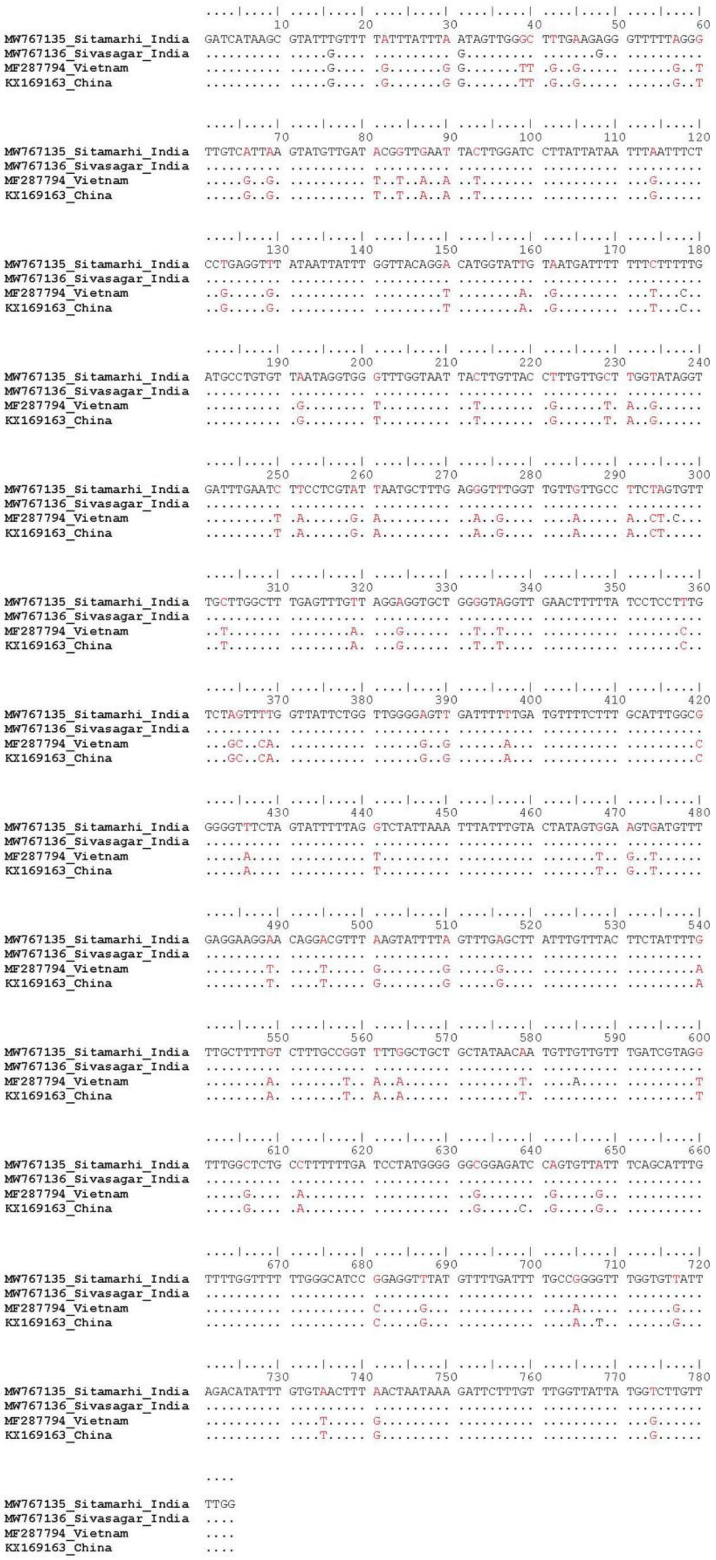

Appendix 2 Figure 3. Alignment of the translated cox1 gene region highlighting genetic variations (red) between *F. buski* isolates from India and from China and Vietnam.
